# The Antecedents and Consequences of Psychological Safety in Airline Firms: Focusing on High-Quality Interpersonal Relationships

**DOI:** 10.3390/ijerph17072187

**Published:** 2020-03-25

**Authors:** Kwang-Ho Lee, Sunghyup Sean Hyun, Haeik Park, Kwangyong Kim

**Affiliations:** 1 Department of Management, Ball State University, 2000 W. University Avenue, Muncie, IN 47306, USA; klee2@bsu.edu; 2 School of Tourism, Hanyang University, 17 Haengdang-dong, Seongdonggu, Seoul 04763, Korea; 3 Department of Hospitality and Tourism Management, Purdue University Fort Wayne, Fort Wayne, IN 46805, USA; parkh@pfw.edu; 4 Korean Standards Association, 5, Teheran-ro 69-gil, Gangnam-gu, Seoul 04763, Korea; kimky@ksa.or.kr

**Keywords:** high-quality interpersonal relationships, psychological safety, learning from failures, creative self-efficacy, creative work involvement

## Abstract

A comprehensive review of the literature on service creativity revealed the necessity to expand the line of creativity-based research in the service-driven industry. It also called for the creation of a survey instrument that entails high-quality interpersonal relationships, psychological safety, and learning from failures, by including two creativity-related constructs, namely, creative self-efficacy and employees’ creative work involvement to the model. The current study aimed; (a) to assess the validity and reliability of measurement models; and (b) to empirically examine the integrated proposed model consisting of salient constructs. A convenience sample of 341 airline employees responded to a self-report questionnaire that was developed using the steps of researchers’ in a comprehensive literature review and refined based on the feedback provided by a panel of five professionals who had worked in airline firms. The resultant data were subjected to exploratory factor analysis (EFA), confirmatory factor analysis (CFA), second-order CFA, and structural equation modeling (SEM) using version 23.0 of AMOS. The results showed that high-quality interpersonal relationships positively influenced psychological safety, which in turn, positively influenced learning from failures and creative self-efficacy. Further, learnings from failures positively influenced creative self-efficacy but not employees’ creative work involvement. Finally, both psychological safety and creative self-efficacy positively influenced employees’ creative work involvement. These findings have significant implications for human resource management practices that aim to promote the creative involvement of airline employees.

## 1. Introduction

Academics and practitioners have acknowledged that employee creativity plays a key role in, not only retaining loyal customers, but also sustaining an organization’s competitive positions [1,2]. Accordingly, service organizations urge their employees to be involved in service innovation and creativity, at both the individual and the group level [3,4]. Such practices represent a practical application of prominent creativity theories in service-driven industries (e.g., airline firms).

Organizational theories (e.g., interorganizational relations theory and organizational creativity theory) emphasize that interpersonal relationships among employees play an important role in the service creativity of a complex organizational system. The primary aspect of these theories suggests that those who value safety in interpersonal domains (i.e., low risk-takers) are likely to exhibit creative behaviors at work [5,6]. Accordingly, an empirical model that is based on existing theoretical literature on interorganizational relations and organizational creativity can be developed; specifically, this model can serve as a framework within which the role that employee relationships and organizational learning experiences play in the service creativity of employees can be situated.

An integrated review of literature, in the field of service creativity, sheds light on the following constructs: High-quality interpersonal relationships [3], psychological safety [3], learning from failures [7], and creative self-efficacy [8]. A recent study, which adopted a novel paradigm on innovation and creativity in service firms, showed that team-based relational ties (which are analogous to high-quality interpersonal relationships) are strongly linked to employees’ service innovation behaviors in the airline industry [9]. Accordingly, some studies have highlighted the importance of employee interaction in sharing and resolving service errors and mistakes that occur during service transactions, and consequently enhancing service creativity [10]. These theoretical views have been used to extend models of creativity that entail creative self-efficacy [8] and creative work involvement [4,11] to interpersonal relationship management among employees in hospitality industries [12], which should be particularly valuable for airline firms that rely primarily on team-based service creativity in favor of sustainable business growth [9]. This discussion makes it apparent that it is necessary to integrate high-quality interpersonal relationships, psychological safety, learning from failures, and creative self-efficacy, in order to nurture the service creativity of employees who work in airline firms.

Previous studies have developed and tested the relationships between high-quality interpersonal relationships, psychological safety, and learning from failures, using samples of employees that represent diverse industries. However, they did not examine the predictive role that creative self-efficacy plays in employees’ creative engagement [13]. Employees’ creative self-efficacy (i.e., confidence, ability, efficacy) predicts creative outcomes (e.g., creative work involvement [14,15]. Therefore, hospitality firms take great efforts to leverage their employees’ creative self-efficacy. However, few studies have explored how creative self-efficacy influences the relationship between high-quality interpersonal relationships and creative work involvement among airline employees. In an attempt to expand this line of research, we sought to address the following theoretical and empirical question: How can the creative self-efficacy and creative work involvement of employees be situated within an integrated creativity model? Specifically, a comprehensive review of the literature on service creativity revealed the necessity to expand the line of creativity-based research in the service-driven industry and called for creation of a survey instrument that entails high-quality interpersonal relationships, psychological safety, and learning from failures, by including two creativity-related constructs, namely, creative self-efficacy and employees’ creative work involvement to the model. Therefore, the current study aimed to; (a) assess validity and reliability of measurement models; and (b) empirically examine the integrated proposed model consisting of salient constructs in the context of airline firms. The results of the study were intended to offer managerial guidelines regarding the strategic human resource management (HRM) practices of airline firms that can promote employees’ work involvement.

## 2. Theoretical Background and Hypotheses

### 2.1. Employees’ Creative Work Involvement

In recent years, the concept of creative work involvement has become increasingly significant since creativity is considered to be a key factor for achieving the competitive performance and productivity in service innovation [16]. According to [17], creative work involvement refers to “the extent to which an employee engages his or her time and effort resources in creative processes associated with work” (p. 36). As a key construct in creating valued ideas at work, creative work involvement is a solid indicator that predicts if an individual will engage in creative tasks at work [18]. As such, creative involvement is a subjective tool that can measure the extent to which individuals are involved in creative tasks [4,19]. The rationale and theoretical foundations of creative work involvement have urged hospitality researchers to do the following: gauge the degree of airline employees’ efforts to engage in service creativity; explore the psychological factors and the underlying psychological mechanisms that foster the degree of creative work involvement in airline firms; and understand the role of creative work involvement in sustaining the operations of airline firms [9]. Nevertheless, there have been few attempts to extend a holistic model of creative work involvement to airline firms. In accordance with the preceding discussion, the present study regarded employees’ creative work involvement as an indicator of the extent to which employees are directly involved in; (a) solving problems that cause others difficulty, (b) trying out new ideas and approaches to problem-solving, and (c) serving as a good role model of creativity; these factors will ensure that customers experience the creative service of an airline firm. The significant role of employee creativity in enhancing performance and productivity has been proposed by recent studies in hospitality contexts. For example, Ref. [20] service employees’ innovative behavior is regarded as a catalyst for initiating creativity and innovation within the service industry. Along with this, as a new HRM challenge in hospitality firms, an effort to make employees be creative service providers has been a great success in terms of both organizations (e.g., sustaining their businesses) and employees (e.g., fostering self-development, confidence to serve my customers without failure, and empowerment), consequently devoting considerable attention to the role of employees’ creative work involvement in hospitality firms [21]. Therefore, exploring the role of employees’ creative work involvement as the outcome of organizational creativity models may enable airline firms to predict their sustained growth of productivity in the competitive airline market.

### 2.2. High-Quality Interpersonal Relationships

High-quality interpersonal relationships have been conceptualized as a multidimensional construct [13]. Theoretically, high-quality interpersonal relationships act as a crucial facilitator that strengthens organizational functioning by promoting service creativity [22,23]. The theoretical and empirical literature on service creativity indicates that employees who engage in high-quality interpersonal relationships are capable of speaking up and resolving service problems and errors that are encountered during service transactions; these lead to opportunities to develop creative service ideas, skills, and processes [24]. This discussion underscores the need to examine the relationship between high-quality interpersonal relationships and employees’ creative work involvement; the resultant findings can help airline firms promote competitive service innovation.

According to previous findings, high-quality interpersonal relationships consist of the following sub-dimensions, which are regarded as predominant predictors of creative performance:

- Tensility is defined as “a relationship’s capacity level for bending and withstanding strain, accommodating changing conditions and the capacity for bouncing back from difficulties” [25].
- Emotional carrying capacity refers to “a relationship’s capacity level for carrying both positive and negative emotions” [25].
- Openness-based connectivity is regarded as “a relationship’s degree of openness to new ideas and influences, and the capacity to deflect behaviours that hinder generative processes” [25].

Employee creativity literature has demonstrated the robust utility of high-quality interpersonal relationships to both practitioners and academics [26]. The literature also makes it apparent that further high-quality relationships promote desirable organizational outcomes. For example, employees with a high capacity to; (a) bounce back from difficulties, (b) experience both positive and negative emotions, and (c) deflect behaviors that hinder creative processes are likely to actively engage in service-related activities in their organizations [25]. Given the utility of the construct in organizational models, high-quality interpersonal relationships should be measured across the three dimensions that it subsumes, namely, tensility, emotional carrying capacity, and openness-based connectivity. In this regard, it is assumed that the construct of high-quality interpersonal relationships should be examined as a significant motivator of the creative work engagement of airline employees.

The construct of high-quality interpersonal relationships, which consists of the dimensions of tensility, emotional carrying capacity, and openness-based connectivity, has been used to determine the psychological safety that an organization engenders. For example, Ref. [25] found that high-quality interpersonal relationships (as well as its dimensions, namely, emotional carrying capacity, tensility, and connectivity) had a significant positive effect on psychological safety (β = 0.46, *p* < 0.001) during organization learning behavior processes. Similarly, Ref. [13] conducted a series of studies that investigated the relationships between high-quality relationships and psychological safety. They found that high-quality relationships significantly and positively influenced psychological safety (study 1: β = 0.65; study 2: β = 0.72). However, there is a paucity of research on the mechanisms by which the three dimensions of high-quality interpersonal relationships significantly influence psychological safety in hospitality contexts (e.g., airline firms). With regard to the relationships between high-quality interpersonal relationships and psychological safety in airline firms, the following hypothesis was proposed:

**Hypothesis** **1.** *High-quality interpersonal relationships (including the dimensions of tensility, emotional carrying capacity, and openness-based connectivity) will positively influence psychological safety in airline firms.*

### 2.3. Psychological Safety 

Psychological safety is defined as “a shared belief that the team is safe for interpersonal risk taking” [27]. The fundamental function of psychological safety is to uncover one’s beliefs about the feedback of their coworkers and/or their organizations, when they disclose the service failures and errors that occurred during service transactions [28]. For example, those with a higher sense of psychological safety may feel confident about their decisions about confronting customers’ diverse needs, rather than feel fearful about potential service failures [29,30]). Furthermore, those with a sense of fear about their service obligations are likely to experience unwanted negative outcomes [31]. A sense of psychological safety makes it possible for employees to overcome such problems by engendering a sense of self-efficacy when they deal with customers’ special requests in unexpected circumstances.

In psychologically safe workplaces, frontline employees perceive fewer risks about potential service errors. Accordingly, employees can speak up about service failures, and they are willing to discuss the underlying service problems with other coworkers in a shared environment; this allows them to take corrective action without the fear of rejection [32,33]. Despite the significant role that psychological safety plays in airline firms, few studies have examined if psychological safety predicts psychological experiences, such as learning from failures, which are linked to the creative performance of airline employees. In general, traditional service firms (e.g., banks) primarily focus on educating their employees to offer standardized services to all customers so that they can minimize the frequency of service failures, which might be supported by the nature of safety climate (i.e., organizations’ safety-supportive managerial practices) [34]. On the other hand, airline firms have been urged to make their workplaces psychologically safe, particularly when employees experienced service errors and mistakes; this will allow them to speak up about their service errors and mistakes without feeling embarrassed, and consequently achieve successful performance of team-based activities in effective interpersonal relations and communication [9,35]. Therefore, it is necessary for airline firms to examine the role of psychological safety as a key mediator in interpersonal relations, learning, and creativity models.

Organizational theory and research suggest that psychological safety significantly predicts the construct of learning from failures. However, little attention has been paid to the exploration of how learning from failures influences the creative work involvement of airline employees. Organizational researchers have proposed a number of reasons that implicate a positive relationship between psychological safety and learning from failures. For example, [27] proposed that the relationship between psychological safety and work performance should be mediated by learning from failures. Some studies have also demonstrated that psychological safety plays a crucial role in enhancing the degree of learning from failures that service organizations engender [11]. More importantly, Ref. [10] found that, in service firms, psychological safety has a significant positive effect on learning from failures, which in turn was positively related to creative performance. Therefore, we proposed the following hypothesis, which suggests that psychological safety will foster learning from failures among employees of airline firms.

**Hypothesis** **2.** *Psychological safety will positively influence learning from failures in airline firms.*

Although, some studies have proposed a structural relationship between psychological safety and creative self-efficacy, few empirical studies have verified such an association. Creative self-efficacy can be regarded as a consequence of psychological safety, which plays a critical role in organizational learning processes. The authors in [35] conducted a comprehensive review to identify the prominent factors that enhance creative self-efficacy. The researcher found that autonomy (i.e., the freedom to decide how to perform one’s work safely) and support for creativity (i.e., searching for fresh and new ways to solve problems) have the potential to enhance the creative self-efficacy of employees who work in an organization. Furthermore, a significant relationship between psychological safety (i.e., a shared belief) and team efficacy has been found in organizational learning and performance research [27]. Based on this discussion, we predicted that airline employees who feel psychologically safe will exhibit greater creative self-efficacy than employees who feel psychologically unsafe. Therefore, the following hypothesis was formulated:

**Hypothesis** **3.** *Psychological safety will positively influence creative self-efficacy in airline firms.*

The findings of previous studies have suggested a link between psychological safety and employees’ creative work involvement. For example, Ref. [4] found that psychological safety significantly influences the creative work involvement (β = 0.44, *p* < 0.001) of college students. More importantly, Ref. [36] found that psychological safety is positively correlated with creative performance (r = 0.25, *p* < 0.01) in healthcare service organizations. Therefore, we proposed the following hypothesis:

**Hypothesis** **4.** *Psychological safety will positively influence creative work involvement in airline firms.*

### 2.4. Learning from Failures

Learning from failures enables employees to leverage their service skills, ideas, and procedures when they engage in creative problem-solving after experiencing crucial service failures. In service contexts, learning from failures is defined as “organizations in which members, when faced with a problem, not only solve it so the task can successfully be completed, but also work to address the problem’s underlying causes” [13]. The findings of past studies suggest that learning from failures is a reliable and valid construct that has significant implications for organizational learning models [37]. The primary role that learning from failures plays in organizational learning processes includes helping employees identify and correct the problem’s underlying causes, and consequently facilitating creative outcomes (e.g., creativity, innovativeness, and productivity [7,38]. The importance of the organizational paradox has been recognized in general business firms but not by operators of airline firms; this is because they promote service-oriented environments that have a zero-tolerance policy for service failures. In this regard, there is no doubt that learning from failures is a crucial process that promotes creative outcomes among airline employees. Therefore, it is reasonable to assume that the habit of learning from failures can be cultivated among employees of airline firms and that the corresponding construct can be used to develop an extended model of employee service creativity.

The theoretical and empirical contributions of previous studies have shown that there are relationships between learning from failures, creative self-efficacy, and creative work involvement among employees who work in service firms. First, Ref. [39] demonstrated that learning from failures is a significant predictor of employee service creativity. It was concluded that that the learning outcomes of Leader-Member Exchange (LMX; i.e., supervisor’s understanding of employees’ job-related problems and failures) have significant positive effects on creative self-efficacy, and consequently enhances service creativity within an organization [35]. More importantly, Ref. [40] found that employee creative self-efficacy is strongly associated with employee learning orientation (i.e., an individual’s sense of confidence, ability, and efficiency). Therefore, we predicted that airline employees who learn from their failures will exhibit greater creative self-efficacy. Therefore, we proposed the following hypothesis:

**Hypothesis** **5.** *Learning from failures positively influences creative self-efficacy in airline firms.*

The proposed relationship between learning from failures and employees’ creative work involvement has been supported by the empirical findings of studies that have been conducted in diverse service contexts. For example, the significant relationship between learning from failures and employees’ creative work involvement has been found to be mediated by creative self-efficacy in service firms [40]. Similarly, [11] found that learning from failures is a significant predictor of employees’ creative performance. Therefore, we proposed the following hypothesis:

**Hypothesis** **6.** *Learning from failures will positively influence creative work involvement in airline firms.*

### 2.5. Creative Self-Efficacy

The concept of creative self-efficacy originates from the self-efficacy theory that was proposed by [41]; in this study, it refers to the sense of competence (e.g., self-perceptions of creativity) of employees who work in service firms [42]. In this regard, Ref. [8] introduced an integrated conceptualization of creativity and self-efficacy, which is called “creative self-efficacy” and is defined as “a key personal attribute for creativity in the workplace” (p. 1137). Past studies on creative self-efficacy have examined it as a mediator [40], a moderator [43], and as a consequence [8] of variables that creativity models entail. Accordingly, some scholars have recognized that creative self-efficacy is a significant measure of the extent to which an individual believes that he or she has the confidence, ability, and efficacy to produce creative outcomes in hospitality firms [12,15]. They have proposed that individuals with high levels of creative self-efficacy (i.e., confidence, ability, and efficacy to exhibit creativity) are likely to perform innovative and creative tasks in hospitality firms. Despite the significant relationship that has been found between creative self-efficacy and creative performance in diverse service firms, a review of a substantial number of studies on employee creativity has overlooked the direct effect of creative self-efficacy (i.e., self-perceptions of creativity) on the creative work involvement of airline employees. Thus, we proposed that creative self-efficacy will mediate the relationship between learning behaviors and the creative work involvement of airline employees.

Previous studies have proposed that creative self-efficacy is a critical antecedent of creative work involvement [8,44], particularly in hospitality firms [15]. According to [45], there is a strong relationship between creative self-efficacy and creativity among research and development (R and D) employees. Consistent with this finding, Ref. [40] demonstrated that employee creative self-efficacy is positively correlated with employee creativity (r = 0.24, *p* < 0.01). In another longitudinal study, Ref. [46] demonstrated a significant relationship between creative self-efficacy and creative work. Further, Ref. [47] showed that Taiwanese high levels of creative self-efficacy were likely to engage in high levels of innovative work behaviors than their counterparts with low levels of creative self-efficacy. More importantly, Ref. [14] found that employees of hospitality firms (e.g., hotels) who had high levels of creative self-efficacy are more likely to generate new ideas and services than their counterparts who had low levels of creative self-efficacy. Therefore, the following hypothesis was formulated:

**Hypothesis** **7.** *Creative self-efficacy will positively influence creative work involvement in airline firms.*

## 3. Research Methods

### 3.1. The Theoretical Framework

**Figure 1:**
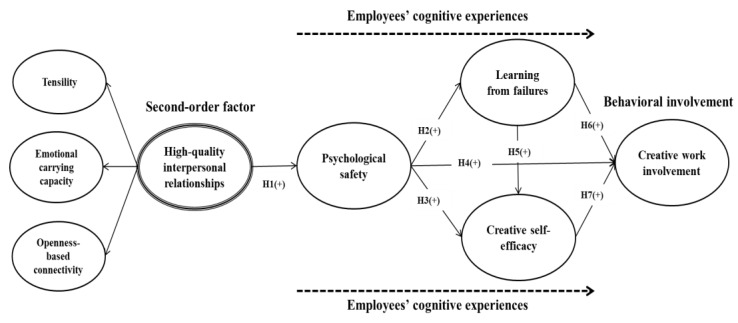
Proposed Study Model.

Figure 1 shows the proposed theoretical framework. On the basis of the preceding discussion on existing theoretical and empirical literature, we tested the structural relationships between high-quality interpersonal relationships (including its three dimensions, namely, tensility, emotional carrying capacity, and openness-based connectivity), psychological safety, learning from failures, creative self-efficacy, and employees’ creative work involvement.

### 3.2. Data Collection

We used measures of high-quality interpersonal relationships, psychological safety, learning from failures, creative self-efficacy, and employees’ creative work involvement that have been used in a wide range of organizational research studies. The measurement scales employed a seven-point Likert-type scale to assess organizational attributes as it has been found to have acceptable psychometric properties in previous organizational research studies [48]. Therefore, responses to all scale items were measured on a seven-point Likert-type scale that ranged from “strongly disagree” (1) to “strongly agree” (7). The resultant scores were used to test the linear relationships between the variables that constituted a structural model (see Table 3).

Previous studies have used a range of different measurement strategies to operationalize organizational variables. Accordingly, in this study we aimed to; (a) test the applicability and relevance of commonly used assessments in the present study; and (b) address the need for a parsimonious set of measures. Hence, the present study employed twelve items to measure high-quality interpersonal relationships (i.e., six items to measure tensility, three items to measure emotional carrying capacity, and three items to measure openness-based connectivity [25]. A panel of five professionals who obtained a doctoral degree by researching airline and/or human resource management in hospitality reviewed the items and found them to be appropriate for use with employees of airline firms. Psychological safety has been measured using three, five, and six items in past organizational research studies. For the sake of parsimony, a three-item measure of psychological safety [13], a five-item measure of learning from failures [46,49], a three-item measure of creative self-efficacy [8], and a seven-item measure of employees’ creative work involvement [17] were used. The questionnaire also included questions that required demographic information such as the respondent’s gender, age, education level, job position, employment status, and work experience.

Prior to data collection, the first version of the questionnaire was reviewed by a panel of five professionals who have at least ten years of experience in the airline industry in order to determine if the selected items are relevant to the respective workplace (i.e., airline firms). The panelists’ feedback did not indicate any concerns about the use of all the aforementioned items in the proposed model. However, some revisions regarding the questions requiring demographic information (e.g., job position and job status) were made to the questionnaire. After completing this procedure, the final questionnaire was distributed to a convenience sample of 500 employees who worked at different international airline firms (e.g., Asiana Airlines, China Eastern Airlines, Japan Airlines, Korea Air, Qatar Airways, and Vietnam Airlines). The respondents could either, respond to a pen-and-pencil version of the self-administrated questionnaire or access an online survey link (i.e., surveymonkey.com) that hosted the survey. Specifically, general managers (or HR managers) of these airline firms were asked to share both online and offline versions of the surveys with their service employees (i.e., cabin attendants and ground service staff). A total of 350 usable responses were subjected to statistical analysis.

We tested the proposed hypotheses using structural equation modeling (SEM). However, using the data screening process of missing data, outliers (z-scores > 3.29), and multivariate normality (Maha-lanobis distance values) [50], nine outliers were detected and excluded. Finally, a total of 341 valid responses were subjected to exploratory factor analysis (EFA), confirmatory factor analysis (CFA), second-order CFA, and SEM using version 23.0 of AMOS.

## 4. Results

### 4.1. Respondent Characteristics

A demographic profile of the respondent characteristics is shown in Table 1. Of the 341 respondents, a majority of them were female (n = 274; 81.8%). Approximately 83% of the respondents had an associate’s degree or a higher degree, and 77% (n = 262), and 23% (n = 79) of the respondents were regular and temporary employees, respectively. In terms of job position, 67% and 33% were cabin attendants (n = 225), and ground service members (n = 112), respectively. Finally, the mean age and tenure of the sample were 31 years and 6.5 years, respectively.

### 4.2. The Exploratory and Conformatory Factor Analyses of Salient Constructs

Behavioral researchers have been urged to implement exploratory factor analysis (EFA), in order to determine if expected factor and/or scale structure is appropriate prior to testing confirmatory factor analysis (CFA), consisting of one or more scales [51,52]. Following this recommendation, an exploratory factor analysis was firstly conducted with the 30 items using principal axis factoring extraction (in conjunction with oblique rotation), specifying seven factors to be extracted and keeping items with a factor loading of over 0.40. The results of the EFA showed that one item in the factor of learning from failures (see Table 3) had an insufficient factor loading (i.e., lower than 0.4; [2]; therefore the one item was deleted.

Another EFA was then conducted on the remaining 29 items using the same method. The results of the Kaiser–Meyer–Olkin (Bartlett’s tests) revealed that the Kaiser–Meyer–Olkin (KMO) overall measure of sampling adequacy was 0.91 (Chi square value = 6699.53, df = 406, *p* < 0.00). The communalities were found to range from 0.51 to 0.78. Therefore, the results of the EFA revealed that seven factors derived from 29 items explained 69% of the variance in the data [53], suggesting the use of the 7-factor model for testing confirmatory factor analysis (CFA) in a comprehensive structural model (see Table 3).

Prior to exploring the unidimentionality, validity, fitness of the proposed model, the present study tested whether high-quality interpersonal relationships should be utilized as a second-order factor structure, consisting of the subdimensions of tensility, emotional carrying capacity, and openness-based connectivity, which have a significant effect on the outcomes that a comprehensive model of service creativity entails. According to [25], high-quality interpersonal relationships can be conceptualized as a multidimensional construct that includes tensility, emotional carrying capacity, and openness-based connectivity. They have verified the robustness of the construct of high-quality interpersonal relationships in organizational research. Thus, this study aimed to reconceptualize the hree dimensions as a single construct (i.e., high-quality interpersonal relationships) in the proposed model. To examine the extent to which a hypothesized model fits the observed data, a series of CFAs were conducted. One might argue that the multiple dimensions of high-quality interpersonal relationships, rather than a unitary factor, must be included in the proposed research framework. However, a second-order factor (i.e., the three first-order factors load onto a single second-order factor) can be used to test the proposed hypotheses. For this purpose, we used the chi-square test and fit indices such as comparative fit index (CFI), Tucker-Lewis index (TLI), normed fit index (NFI), and root mean square error of approximation (RMSEA; [47,48,49]). Accordingly, the resultant estimates for the second-order CFA model were based on the three dimensions, namely, tensility, emotional carrying capacity, and openness-based connectivity being loaded onto high-quality interpersonal relationships and suggested that the data fit the model well: χ2 = 168.21 (df = 47, *p* < 0.01), TLI = 0.94, NFI = 0.94, and RMSEA = 0.08 (see Table 2). The use of higher-order factor models (i.e., parsimonious frameworks) should be recommended, even though the model fits of second-order factor models are lower than that of first-order correlated models in comprehensive multidimensional frameworks [54]. Therefore, these findings confirmed that the unitary construct of high-quality interpersonal relationships, which consists of tensility, emotional carrying capacity, and openness-based connectivity, should be utilized to test the proposed hypotheses.

As a next step, a CFA was conducted to confirm the measurement model using maximum likelihood estimation method and in accordance with the recommendations of [50]. The results of the CFA revealed that one item in the factor of learning from failures (see Table 3) had insufficient factor loadings (i.e., lower than 0.5; [53]. Therefore, the single item was deleted. Subsequently, another CFA was conducted to examine the underlying structures (i.e., unidimensionality) of all the measurement variables that were included in the model. The resultant fit indices of the model were satisfactory: χ2 = 460.920, df = 160, χ2/df = 2.88, CFI = 0.93, TLI = 0.93, and RMSEA = 0.07. The result revealed that the data supported the proposed measurement model.

The construct validity and reliability of the measurement scales are presented in Table 3 and Table 4 With regard to convergent validity, all CFA factor loadings ranged from 0.69 to 0.91 (see Table 3) and the average variance extracted (AVE) value of each construct ranged from 0.51 to 0.74 (i.e., >0.5; see Table 4); these findings suggest that the constructs have adequate convergent validity [46]. In addition, as presented in Table 4, AVE values ranged from 0.71 to 0.86 and all values exceeded the square of the correlations; these findings suggest that all the constructs that were included in the model have good discriminant validity.

### 4.3. Results Pertaining to the Research Hypotheses

**Figure 2:**
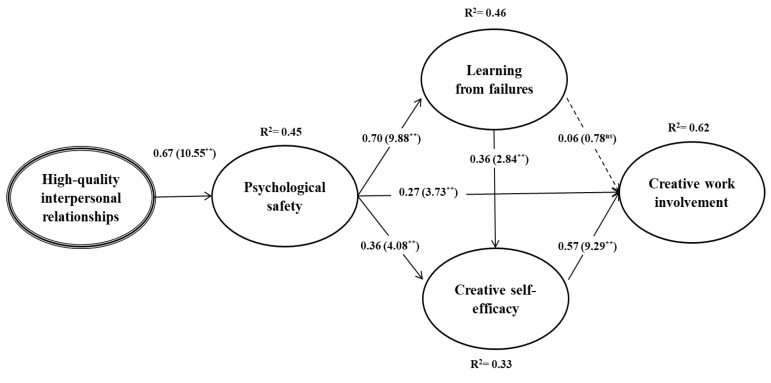
The results of structural equation modelling for the extended model of creativity. Note: ns = not significant, ** *p* < 0.01. The numbers that are presented in parentheses are t-values, and those that are outside the parentheses are standardized path coefficients. The dotted line indicates a non-significant effect.

Table 5 and Figure 2 show the results of SEM for the proposed model. The extent to which the model fit the data was assessed using various goodness-of-fit indices. Overall, the results revealed a reasonable fit [50] χ2 = 492.622, df = 161, χ2/df = 3.06, CFI = 0.93, TLI = 0.91, and RMSEA = 0.07. Specifically, the results supported all hypotheses except H6. First, high-quality interpersonal relationships (β = 0.67, *t* = 10.55, *p* < 0.01) had a significant positive effect on psychological safety. Second, psychological safety significantly and positively influenced learning from failures (β = 0.70, *t* = 9.88, *p* < 0.01), creative self-efficacy (β = 0.36, *t* = 4.08, *p* < 0.01), and employees’ creative work involvement (β = 0.27, *t* = 2.84, *p* < 0.01). Finally, creative self-efficacy had a significant positive effect on employees’ creative work involvement (β = 0.57, *t* = 9.29, *p* < 0.01). In sum, the results provide support for H1, H2, H3, H4, H5, and H7, but not H6. Additionally, Table 5 presents the results that pertain to the indirect relationships between the proposed constructs. Specifically, the results of the bootstrap test at a 99% confidence interval with 10,000 bootstrap samples [56] confirmed the existence of positive and significant indirect effects for psychological safety between high-quality interpersonal relationships and learning from failures (standardized indirect effect = 0.55, *p* < 0.01) and between high-quality interpersonal relationships and creative self-efficacy (standardized indirect effect = 0.44, *p* < 0.01). The indirect results also showed a positive and significant indirect effect for learning from failures between psychological safety and creative self-efficacy (standardized indirect effect = 0.14, *p* < 0.05), a positive and significant indirect effect for learning from failures between psychological safety and creative self-efficacy (standardized indirect effect = 0.14, *p* < 0.05), and a positive and significant indirect effect for creative self-efficacy between learning from failures and creative work involvement (standardized indirect effect = 0.15, *p* < 0.05). The results also showed significant and positive indirect effects for learning from failures and creative self-efficacy between psychological safety and creative work involvement (standardized indirect effect = 0.26, *p* < 0.01). Additionally, there were significant and positive indirect effects for psychological safety, learning from failures, and creative self-efficacy between high-quality interpersonal relationships and creative work involvement (standardized indirect effect = 0.49, *p* < 0.01).

## 5. Discussion 

A deeper understanding of employees’ creative work involvement plays a critical role in the development of the innovation management practices of airline firms, which should be particularly valuable for airline firms that depend significantly on team-based service creativity in favor of sustainable business growth. Accordingly, the findings of the present study shed light on the integrated theoretical model, which includes the following constructs: high-quality interpersonal relationships, which consists of three dimensions, namely, tensility, emotional carrying capacity, and openness-based connectivity [17], psychological safety [13], and learning from failures [49]. We achieved this by adding the constructs of creative self-efficacy [46] and employees’ creative work involvement [57] to the aforementioned comprehensive model that has not been previously applied to hospitality contexts. Although, some studies have found that high-quality interpersonal relationships influence learning from failures and psychological safety in service organizations [13], they have not attempted to examine the creativity-relevant constructs of creative self-efficacy and creative work involvement in hospitality firms (i.e., airline firms). Therefore, understanding the structural relationships among salient creativity constructs (i.e., creative self-efficacy and creative work involvement) is significant. Specifically, creative work involvement can serve as a critical indicator of the degree to which employees exhibit creative behaviors; these behaviors will ensure that customers experience the creative service of airline firms [4,19].

Our findings support the theoretical contention of the proposed holistic model that employees’ high-quality interpersonal relationships boost the magnitude of their creative work involvement by fostering psychological safety, learning from failures, and creative self-efficacy [11,13,25,46]. These results are consistent with the basic tenets of interorganizational relations theory and organizational creativity theory, which suggest these behaviors will ensure that customers experience the service innovation of airline firms that organizational efforts to make the work environment safe, and to reinforce employees’ interpersonal exchanges and relationships help employees create novel ideas and services [6]. In sum, it is necessary for hospitality researchers to extend the application of the following comprehensive model, which is based on organizational theories (e.g., interorganizational relations theory, organizational creativity theory), to airline firms in favor of the structural relationships between high-quality interpersonal relationships, psychological safety, learning from failures, creative self-efficacy, and creative behavior (creative work involvement).

## 6. Theoretical Implications

Organizational theorists have acknowledged the significance of employees’ creative involvement at work [11]. However, few studies have identified the mediators that influence the relationship between high-quality interpersonal relationships and employees’ creative work involvement in hospitality firms. Thus, the present study attempted to build an extended theoretical model that explores the structural relationships between high-quality interpersonal relationships, psychological safety, and learning from failures using samples of airline employees.

Specifically, a significant relationship emerged between high-quality interpersonal relationships and psychological safety. This result is consistent with the previous findings that a relationship between high-quality interpersonal relationships and psychological safety underlies organizational learning processes [13,25]. However, there is little consensus about whether the dimensions of high-quality interpersonal relationships, namely, tensility, emotional carrying capacity, and openness-based connectivity significantly enhance the degree of psychological safety in airline firms. Our findings suggest that high-quality interpersonal relationships and psychological safety are closely related; therefore, these findings can help airline firms create an environment that employees perceive to be psychologically safe (i.e., low interpersonal threat). The result can be supported by the paradigm of service-driven organizations in favor of interpersonal relationships and risk taking, which provides creativity-supporting collaborative working environments. in order to build and maintain a self-developed creativity mechanism that support productivity in team-based tasks. Consequently, organizational theorists should recognize the effect that the three dimensions of high-quality interpersonal relationships, namely, tensility, emotional carrying capacity, and openness-based connectivity, have on the psychological safety of airline employees.

The results suggest that there are structural relationships between psychological safety, learning from failures, creative self-efficacy, and employees’ creative work involvement. The finding that psychological safety is related to learning from failures (β = 0.70, *t* = 9.88) is consistent with previous findings that psychological safety has a significant effect on learning from failures among employees of service firms [10]. This suggests that airline employees, with high levels of psychological safety, feel confident about their decisions rather than fearful about potential service failures when they confront customers’ diverse needs. The results can be explained by the fact that hospitality firms focus on the management orientation of service creativity and innovation by, not only strengthening interpersonal relationships among employees, but also facilitating a shared belief in mitigating perceived risk taking. With regards to the measurement scale of psychological safety, of the different scales (i.e., three-item scale, five-item scale, or six-item scale) that have been used to assess psychological safety in organizational research studies, the scores yielded by the three-item measure significantly predicted the cognitive outcomes of airline employees. In this regard, a majority of past studies have employed five-item, six-item, and eight-item scales to assess psychological safety and examine if it predicts learning from failures in different organizational contexts. Therefore, the findings of the present study suggest that the three-item measure of psychological safety should be utilized to test the cognitive consequences of learning behavior models in airline firms.

The emergent relationship between psychological safety and creative self-efficacy (β = 0.36, *t* = 4.08) implies that airline employees with high levels of psychological safety are likely to exhibit high levels of creative self-efficacy (i.e., beliefs about one’s confidence, ability, and efficiency). This result is consistent with previously identified associations between autonomy (i.e., the freedom to decide how to perform one’s work safely) and creative efforts at work [35]. This finding is also line with a previous study [34], positing that employees who work in hospitality firms should not be blamed for their service failures, errors, and mistakes; this provides supports the observed link between psychological safety (i.e., interpersonal forgiveness and reconciliation in workplace relationships) and the creative self-efficacy of airline employees. Accordingly, the finding implies that psychological safety (i.e., interpersonal forgiveness and reconciliation) should be used as an indicator of the creative self-efficacy (i.e., confidence, ability, and efficiency) of airline employees.

The psychological safety and employees’ creative work involvement (β = 0.27, *t* = 2.84) indicates that airline employees with high levels of psychological safety are more likely to exhibit creative behaviors. This finding is consistent with the theoretical contention that hospitality firms must encourage their employees to be engaged in generating and improvising upon innovative services during service errors and mistakes in creative processes associated with work [34]. Although, a significant association emerged between psychological safety and employees’ creative work involvement, there is a paucity of research findings that delineate how psychological safety influences the creative work involvement of airline employees in airline firms. In this regard, it is important to focus on the critical role that learning from failures plays in fostering the creative work involvement of airline employees.

The result that learning from failures has an indirect effect on employees’ creative work involvement (i.e., through creative self-efficacy) is inconsistent with previous findings that learning from failures has a direct effect on creative performance [11]. According to [40], learning from failures has an indirect effect on employees’ creative work involvement that is mediated by creative self-efficacy in service organizations. Such an observation is consistent with the salient role that creative self-efficacy was found to play in the relationship between learning from failures and the creative work involvement of airline employees. This suggests that learning from failures may not directly influence the creative work involvement of airline employees. In other words, the effect of learning from failures on employee creativity performance (e.g., creative work involvement) is fully mediated by creative self-efficacy. In accordance with the critical role that learning from failures was believed to play in organizational learning processes, learning from failures has been found to help employees identify and correct the problem’s underlying causes, and consequently produce creative outcomes (e.g., creativity, innovativeness, and productivity; [7,38]. Understanding such organizational paradoxes should not be limited to hospitality firms; indeed, they have been neglected by operators of airline firms due to their pursuit of service-oriented environments promoting no service failure group. In this regard, there is no doubt that learning from failures is a crucial process that promotes creative outcomes among airline employees.

As expected, a significant relationship emerged between creative self-efficacy and creative work involvement (β = 0.57, *t* = 9.29). This finding implies that creative self-efficacy predicts the creative work involvement of airline employees. This finding also suggests that creative self-efficacy may foster a better understanding of the creative work involvement and may lead to service creativity in improving organizational performance (e.g., creativity). In recent years, the literature in the field of organizational behavior has focused on self-efficacy as a global construct, in order to develop strategic HRM practices. However, creative self-efficacy is a term that was coined by [8] to refer to employees’ self-perceptions (i.e., confidence, ability, and efficiency) of creativity in their respective organizations [8,42]. Some studies have found that service-oriented employees, who work in hospitality industries, are more likely to be involved in creative work than their counterparts who work in business firms that focus primarily on physical products [57]. Therefore, it is noteworthy that creative self-efficacy is a prominent psychological factor that yields creativity-relevant outcomes (e.g., creative work involvement) among airline employees. Further, this finding underscores the importance of establishing safe and harmonious workplaces that offer their employees the opportunities to generate new service ideas and actions.

## 7. Practical Implications

Our findings have managerial implications that have the potential to inform the development of HRM practices in airline firms. First, in accordance with the direct relationship that emerged between high-quality interpersonal relationships (i.e., tensility, emotional carrying capacity, and openness-based connectivity) and psychological safety, strategic HRM practices should seek to maintain harmonious relationships between employees. More specifically, airline firms should provide management training sessions that encourage team leaders to (i) create flexible environments in which employees adopt a “Don’t be afraid to speak up” attitude, (ii) discuss failures issues with their coworkers in a confidential manner and effectively resolve them without causing further problems, and (iii) help employees develop co-created service skills. Second, the emergent relationship between psychological safety and learning from failures implies that airline firms should urge their employees to immediately respond to the underlying causes of a service failure, in order to meet the diverse needs of customers in an innovative manner. Third, the relationships that learning from failures, shared with creative self-efficacy and creative work involvement, and the relationship that emerged between creative self-efficacy and creative work involvement, should inform the development of management practices such as creativity and innovation-oriented employee involvement programs. For example, airline firms need to maintain a highly interpersonal organizational atmosphere (e.g., hierarchical communication climate is associated with shared communication and forgiveness climate), which makes their employees aware that they can speak up about their service failures, and share the underlying problems and errors with their coworkers in psychologically safe environments. In addition, airline firms should tolerate employees’ service failures to facilitate experience-based learning, which is a necessary challenge that may ultimately enhance the creative involvement of employees of airline firms. To sum up, industry practitioners need to aware of the fact that employees’ creative work involvement comes up with competitive performance and productivity in the service-drive industry (i.e., airline firms). As a key challenge for developing innovative HR practices, airline firms need to reform their efforts to make employees feel a sense of interpersonal harmony in team-based service delivery for customers who rely heavily on creative services in consumption. Importantly, such a practical challenge may produce beneficial outcomes for both airline firms and employees. Form an organizational perspective, airline firms can utilize the measure of employees’ creative work involvement as an index for gauging their business success in sustainability. For employees, those who are actively involved in creative service delivery are able to achieve self-development, such as new skills for team-based service collaboration, the confidence and ability/efficacy of “I can do for my customers”, and empowerment. Consequently, a better understanding of employees’ creative work involvement may allow airline firms to achieve the continued growth of performance in service innovation.

## 8. Limitations and Future Research

This study has some limitations. First, the present study tested the validity of the proposed research model using data obtained from Korean employees who work in eight airline firms in Asia. Cross-sectional data that entails a representation of diverse airline segments (e.g., low-cost airlines and full-service airlines) and different ethnicities and nationalities may enhance the generalizability of the study findings. Such a diverse data set would improve the statistical power and allow us to examine cross-level interactions. Second, [49] used the five-item measure of learning from failures that was developed by [46], in order to test the measurement model of learning from failures using 106 responses. The results suggested that one of the items (i.e., “When a problem concerning a lack of required resources for completing a task is being raised, I provide an immediate solution, but also inform the management and the relevant department about the problem”) should be discarded because it did not yield a sufficient factor loading (0.60; i.e., it is below the 0.70 cutoff that was computed using 106 responses; [53]). Consistent with such a finding, this item was excluded from the scale in this study; indeed, the factor loading was below the 0.5 cutoff 341 responses [53]. This suggests that the four-item measure of learning from failures should be used to predict the creative outcomes of airline employees. Given the need for a parsimonious set of measures, future researchers should test the concrete reliability and validity of a four-item measure of learning from failures that is designed for use in airline firms (see Table 3). Finally, it appears that employees’ creative work involvement can be used to predict the extent to which customers experience service innovation. However, this study did not examine the role that employees’ creative work involvement plays in customers’ experience of service innovation. Therefore, future researchers must use new methodological approaches to test the proposed correlations between employees’ creative work involvement and customers’ perceptions of the creative service of airline employees.

## Figures and Tables

**Table 1:** Demographical profile of the study participants.

Variable	Frequency (%)
Gender (n = 335) ^a^	
Male	61(18.2)
Female	274(81.8)
Educational level (n = 336) ^b^	
High school degree	2(0.6)
Associate’s degree	71(21.1)
Bachelor’s degree	241(71.7)
Graduate degree	22(6.6)
Job Position (n = 337)	
Cabin attendants	225(66.8)
Ground service staff	112(33.2)
Employment status (N = 341)	
Regular	262(76.8)
Temporary	79(23.2)
Mean age in years (SD; N = 341)	31(8.45)
	6.5(6.53)

Note: ^a^ 6 missing values, ^b^ 5 missing values.

**Table 2:** Results of the confirmatory factor analysis for the high-quality interpersonal relationship models.

Model	Absolute Fit Indices		Incremental Fit Indices		Parsimonious Fit Indices	AVE	CR
	GFI (>0.90)	RMSEA (<0.08)	TLI (>0.90)	NFI (>0.90)	χ2/df (<5.0)		
One factor (TEN + ECC + OBC)	0.862	0.13	0.870	0.891	319.48/48 = 6.66	0.55	0.93
Two factors (TEN/ECC + OBC)	0.877	0.12	0.889	0.907	273.60/47 = 5.82	0.62/0.52	0.91/0.87
Two factors (ECC/TEN + OBC)	0.888	0.11	0.898	0.913	255.08/47 = 5.43	0.60/0.56	0.82/0.92
Two factors (OBC/TEN + ECC)	0.911	0.09	0.925	0.932	200.67/47 = 4.27	0.64/0.57	0.84/0.92
Three factors (TEN/ECC/OBC)	0.941	0.08	0.951	0.952	141.78/45 = 3.14	0.62/0.60 /0.64	0.91/0.82 /0.84
Second-order factor (TEN/ECC/OBC)	0.931	0.08	0.941	0.943	168.21/47 = 3.56	0.69	0.95

Note: TEN = tensility; ECC = emotional carrying capacity; OBC = openness-based connectivity; AVE = average variance extracted; CR = composite reliability; GFI = goodness of fit index; CFI = comparative fit index, TLI = Tucker-Lewis index, NFI = normed fit index, and RMSEA = root mean square error of approximation.

**Table 3:** The exploratory and confirmatory factor analyses of salient constructs.

Constructs (Cronbach’s α)	Items	Factor Loadings Exploratory Results	Completely Std. Coeff. Confirmatory Results
	Tensility [25]		
High-quality interpersonal relationships (0.86)	TEN1: My co-workers and I cope well with the conflicts we experience at work	0.61	0.86 **
	TEN2: My co-workers and I cope well with the tensions we experience at work		
	TEN3: My co-workers and I cope well with the pressures we experienced at work		
	TEN4: Even during times of stress and pressure, my co-workers and I always manage to find effective solutions		
	TEN5: Even when we are very busy and under pressure at work, my co-workers and I maintain good relationships		
	TEN6: After my co-workers and I overcome major crises and periods of tension together, our relationships are stronger, not weaker		
	Emotional carrying capacity [22,55]		
	ECC1: My co-workers and I do not have any difficulty expressing our feelings to one other	0.56	0.80 **
	ECC2: My co-workers and I are not afraid to express our unpleasant feelings at work		
	ECC3: Whenever anyone at work expresses an unpleasant feeling, he/she always does so in a constructive manner		
	Openness-Based Connectivity [25]		
	OBC1: We are always open to listening to our co-workers’ new ideas	0.57	0.79 **
	OBC2: We are very open to diverse influences, even if they come from unconventional sources, such as new employees, customers, etc.		
	OBC3: We know how to accept people who are different		
Psychological safety (0.85) [13]	PSS1: If I make a mistake in our airline firm, it is often held against me (reverse scored item)	0.84	0.84 **
	PSS2: It is safe to take a risk in our airline firm	0.81	0.83 **
	PSS3: No one in our airline firm would deliberately act in a way that would undermine my efforts	0.76	0.77 **
Learning from failures (0.80) [49,53]	LFF2: When I make a mistake, my team members in this airline company talk to me, not for the purpose of blaming me, but rather for the value of learning	0.63	0.71 **
	LFF3: When I make a mistake, I inform the relevant manager to enable others to learn from it	0.81	0.72 **
	LFF4: A question such as “why do we do the things as such” is fully appreciated in our airline firm	0.66	0.66 **
	LFF5: In our airline firm, I am encouraged to ask questions such as “is there a better way to provide the service”	0.79	0.76 **
Creative self-efficacy (0.82) [8]	CSE1: I have confidence in my ability to solve problems creatively	0.73	0.85 **
	CSE2: Many times I have proved that I can cope with difficult situations	0.72	0.88 **
	CSE3: I know I can efficiently solve even complicated problems	0.68	0.85 **
Employees’ creative work involvement (0.92) [17]	CWI1: I demonstrate originality at my work	0.75	0.80 **
	CWI2: I take risks in terms of producing new ideas in doing my job	0.78	0.70 **
	CWI3: I solve problems that had caused others difficulty	0.74	0.77 **
	CWI4: I try out new ideas and approaches to problems	0.79	0.80 **
	CWI5: I identify opportunities for new ideas/services	0.78	0.85 **
	CWI6: I generate novel, but operable work-related ideas	0.78	0.86 **
	CWI7: I serve as a good role model for creativity	0.77	0.77 **

Note: ** *p* < 0.01; Model fit: χ2 = 460.920, df = 160, χ2/df = 2.881, CFI = 0.93, NFI = 0.93, RMSEA = 0.07; Items with insufficient factor loadings (i.e., ≤ 0.4; [47] was deleted).

**Table 4:** Intercorrelations between the salient constructs.

Constructs	1	2	3	4	5
1. High-quality interpersonal relationships (HQIR)	(0.819)				
2. Psychological safety (PSS)	0.529 **	(0.812)			
3. Learning from failures (LFF)	0.617 **	0.547 **	(0.714)		
4. Creative self-efficacy (CSE)	0.467 **	0.453 **	0.446 **	(0.860)	
5. Creative work involvement (CWI)	0.411 **	0.532 **	0.463 **	0.684 **	(0.794)
Mean	4.72	4.44	4.70	4.46	4.18
SD	0.95	1.24	1.08	1.03	0.98
AVE	0.67	0.66	0.51	0.74	0.63
CR	0.86	0.85	0.80	0.90	0.93

Note: Values that are italicized and presented within parentheses represent the square of the AVE value of each construct. ** indicates significant values (*p* < 0.05)

**Table 5:** A summary of the results that were used to test the research hypotheses.

Hypotheses	Direct Paths	Standardized Path Coefficient
Hypothesis 1	High-quality interpersonal relationships → psychological safety	0.67 **
Hypothesis 2	Psychological safety → learning from failures	0.70 **
Hypothesis 3	Psychological safety → creative self-efficacy	0.36 **
Hypothesis 4	Psychological safety → creative work involvement	0.27 **
Hypothesis 5	Learning from failures → creative self-efficacy	0.26 **
Hypothesis 6	Learning from failures → creative work involvement	0.06ns
Hypothesis 7	Creative self-efficacy → creative work involvement	0.57 **
	Indirect paths	
	HQIR → PSS → LFF	0.55 **
	HQIR → PSS → CSE	0.44 **
	PSS → LFF → CSE	0.14 **
	LFF → CSE → CWI	0.15 **
	PSS → LFF → CSE→ CWI	0.26 **
	HQIR → PSS → LFF → CSE→ CWI	0.49 **

Note: ^**^ *p* < 0.01. Model fit: χ^2^ = 492.622, df = 161, χ2/df = 3.06, CFI = 0.93, TLI = 0.91, RMSEA = 0.07. Bootstrapping method was used to estimate the standardized coefficients for the indirect effects.
